# Pharmacology of LRRK2 with type I and II kinase inhibitors revealed by cryo-EM

**DOI:** 10.1038/s41421-023-00639-8

**Published:** 2024-01-23

**Authors:** Hanwen Zhu, Patricia Hixson, Wen Ma, Ji Sun

**Affiliations:** 1 Department of Structural Biology, St. Jude Children’s Research Hospital, Memphis, TN USA; 2 Department of Physics, University of Vermont, Burlington, VT USA

**Keywords:** Structural biology, Post-translational modifications

## Abstract

LRRK2 is one of the most promising drug targets for Parkinson’s disease. Though type I kinase inhibitors of LRRK2 are under clinical trials, alternative strategies like type II inhibitors are being actively pursued due to the potential undesired effects of type I inhibitors. Currently, a robust method for LRRK2–inhibitor structure determination to guide structure-based drug discovery is lacking, and inhibition mechanisms of available compounds are also unclear. Here we present near-atomic-resolution structures of LRRK2 with type I (LRRK2-IN-1 and GNE-7915) and type II (rebastinib, ponatinib, and GZD-824) inhibitors, uncovering the structural basis of LRRK2 inhibition and conformational plasticity of the kinase domain with molecular dynamics (MD) simulations. Type I and II inhibitors bind to LRRK2 in active-like and inactive conformations, so LRRK2–inhibitor complexes further reveal general structural features associated with LRRK2 activation. Our study provides atomic details of LRRK2–inhibitor interactions and a framework for understanding LRRK2 activation and for rational drug design.

## Introduction

Leucine-rich repeat kinase 2 (LRRK2) is a highly pursued drug target for Parkinson’s disease (PD), which is the second most common neurodegenerative disease projected to affect 17.5 million people by 2040^1^. LRRK2 is a 286-kDa protein containing seven domains (ARM, ANK, LRR, ROC, COR, KIN, and WD40) with kinase and GTPase activities^2,3^. LRRK2 plays crucial roles in multiple cellular signaling pathways associated with PD, such as ciliogenesis, mitophagy, autophagy, and mitochondrial homeostasis^2,4,5^. Autosomal dominant LRRK2 mutations with higher kinase activity are leading genetic causes of both familial and sporadic late-onset PD cases^6–8^. On the other hand, LRRK2 knockout is neuroprotective in cell and animal models^9–12^, providing a strong rationale for targeting LRRK2 for PD treatment.

Developing selective inhibitors of LRRK2 has been a major focus in treating LRRK2-associated PD. Various targeting strategies are currently being explored, including ATP-competitive type I and type II inhibitors, LRRK2 dimerization inhibitors, LRRK2 G2019S selective inhibitors, antisense oligonucleotide, proteolysis targeting chimeras (PROTACs), and LRRK2-targeting nanobodies^13–20^. Currently, LRRK2-specific type I kinase inhibitors are available, and type II inhibitors are under active exploration. LRRK2 inhibitors, including DNL201 and DNL151, are currently in clinical trials.

The lack of co-structures of LRRK2 and small-molecule inhibitors largely hinders our understanding of the mode of action (MoA) of known inhibitors and ongoing efforts for structure-based drug discovery. Structural information on drug targets with inhibitors enables medicinal chemists to understand the structure-activity relationship (SAR) and facilitates lead optimization and computation-aided drug discovery. However, experimental methods that enable the determination of high-resolution LRRK2 structures with different types of inhibitors are lacking.

Here we report high-resolution cryo-EM structures of LRRK2 in complex with a panel of small molecules, including two type I (LRRK2-IN-1 and GNE-7915) and three type II inhibitors (rebastinib, ponatinib, and GZD-824). This study established workflows for the structural determination of LRRK2–inhibitor complexes, provided the molecular basis underlying the recognition mechanism of diverse small molecules by LRRK2, and elucidated the common structural features associated with LRRK2 activation.

## Results

### Structural determination of LRRK2–inhibitor complexes

We examined the inhibition effect of small molecules on purified LRRK2 using an in vitro ADP-Glo kinase assay, which monitors the ATP consumption to measure the kinase activity of LRRK2^21,22^ (Supplementary Fig. S1a). Here the cryoEM construct, LRRK2^RCKWm^ (see below for details), is used for the inhibition assay. We selected two type I inhibitors (LRRK2-IN-1 and GNE-7915) and three type II inhibitors (rebastinib, ponatinib, and GZD-824), which inhibit LRRK2 and stabilize it in active-like or inactive conformations, respectively. All these compounds showed pronounced inhibition of LRRK2 (Supplementary Fig. S1b, c). The IC_50_ values differ slightly from what previously has been reported by less than one order of magnitude^23–25^. This observation can be likely explained due to the differences in kinase assays. Type II inhibitors used in this study are less potent than type I inhibitors, as previously reported^15,23,26^. Additionally, the inhibition assay, with the settings in Supplementary Fig. S1a, is amenable to high-throughput drug screening and estimated to have the capacity to test ~100 k compounds per 1 mg purified sample.

**Fig. 1:**
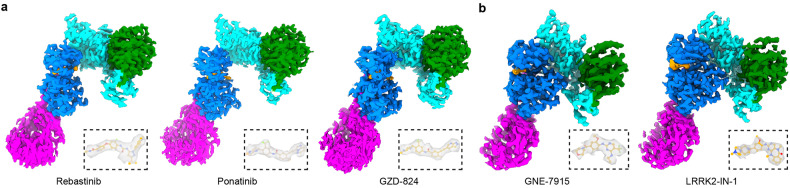
Overall structures of LRRK2^RCKW^ bound to type I or type II inhibitors. **a** Cryo-EM maps of LRRK2^RCKW^ bound to type II inhibitors. Left: LRRK2–rebastinib complex; Middle: LRRK2–ponatinib complex; Right: LRRK2–GZD-824 complex. Only C-terminal catalytic halves of LRRK2 (LRRK2^RCKW^) bound to type II inhibitors are shown for simplicity and better comparison. **b** Cryo-EM maps of LRRK2 bound to type I inhibitors. Left: LRRK2^RCKWm^–GNE-7915 complex; Right: LRRK2^RCKWm^–LRRK2-IN-1 complex. The cryo-EM maps are colored by domain architecture: ROC green, COR cyan, KIN blue, WD40 magenta, inhibitor yellow. Cryo-EM density maps of inhibitors are shown in dashed boxes.

For the structural characterization of LRRK2–inhibitor complexes by single-particle cryo-EM analysis, we applied different experimental strategies for type I and type II inhibitors. Type II inhibitors bind LRRK2 in a kinase-open inactive state, which should resemble what was captured in our previous study^21^. Following a similar purification approach, type II inhibitors were mixed with full-length LRRK2 protein in an ATP-free buffer before cryo-EM analysis. Both LRRK2 monomer and dimer exist in the same grid, and we were able to resolve LRRK2–ponatinib, LRRK2–GZD-824, LRRK2–rebastinib complexes at nominal overall resolutions of 3.4, 3.6, and 3.7 Å, respectively (Fig. 1a; Supplementary Fig. S2 and Table S1).

For type I inhibitors, we designed an LRRK2^RCKW^ construct with M1732R mutation (LRRK2^RCKWm^) based on the following observations and rationales. First, full-length LRRK2 tends to aggregate in the presence of type I inhibitors, likely because the binding of such inhibitors leads to an active-like conformation that exposes a second dimerization interface on the WD40 domain, which is shielded in the inactive state^21,27^. Therefore, we introduced the M1732R mutation, which disrupts COR-B dimerization and LRRK2 filamentation, to prevent protein aggregation^21^. Second, the binding of type I inhibitors could lead to a kinase-closed active-like conformation with flexible N-terminal ARM-ANK-LRR domains, which did not show interaction with the rest of the protein upon activation^28–30^. These domains do not contribute to drug binding but pose challenges for high-resolution structural determination by introducing flexibility upon type I inhibitor binding, so we used the previously reported LRRK2^RCKW^ construct without ARM-ANK-LRR domains^29,30^. We determined LRRK2^RCKWm^ structures with LRRK2-IN-1 and GNE-7915 at overall resolutions of 3.5 and 3.8 Å, respectively (Fig. 1b; Supplementary Fig. S3 and Table S2).

### LRRK2–type II kinase inhibitor complexes

The overall structures of LRRK2–type II kinase inhibitor complexes resemble the inactive state^21^, as type II inhibitors bind LRRK2 in a kinase-open inactive conformation. Structural alignment of LRRK2-alone structure (PDB 7LHW) with LRRK2–ponatinib, LRRK2–GZD-824, and LRRK2–rebastinib complexes results in RMSD of 1.4, 1.4 and 1.6 Å, respectively, suggesting similarity in overall configurations but local differences (Supplementary Fig. S4a). Comparing the drug-bound LRRK2^RCKW^ domains (LRRK2–ponatinib, LRRK2–GZD-824, and LRRK2–rebastinib) with the ATP-bound inactive (LRRK2 alone and LRRK2 in the Rab29–LRRK2 monomer)^21,22^ structures revealed only a small rigid-body movement of the ROC-COR domains relative to the KIN-WD40 domains (Supplementary Fig. S3b), induced by ponatinib, GZD-824, and rebastinib binding.

**Fig. 2:**
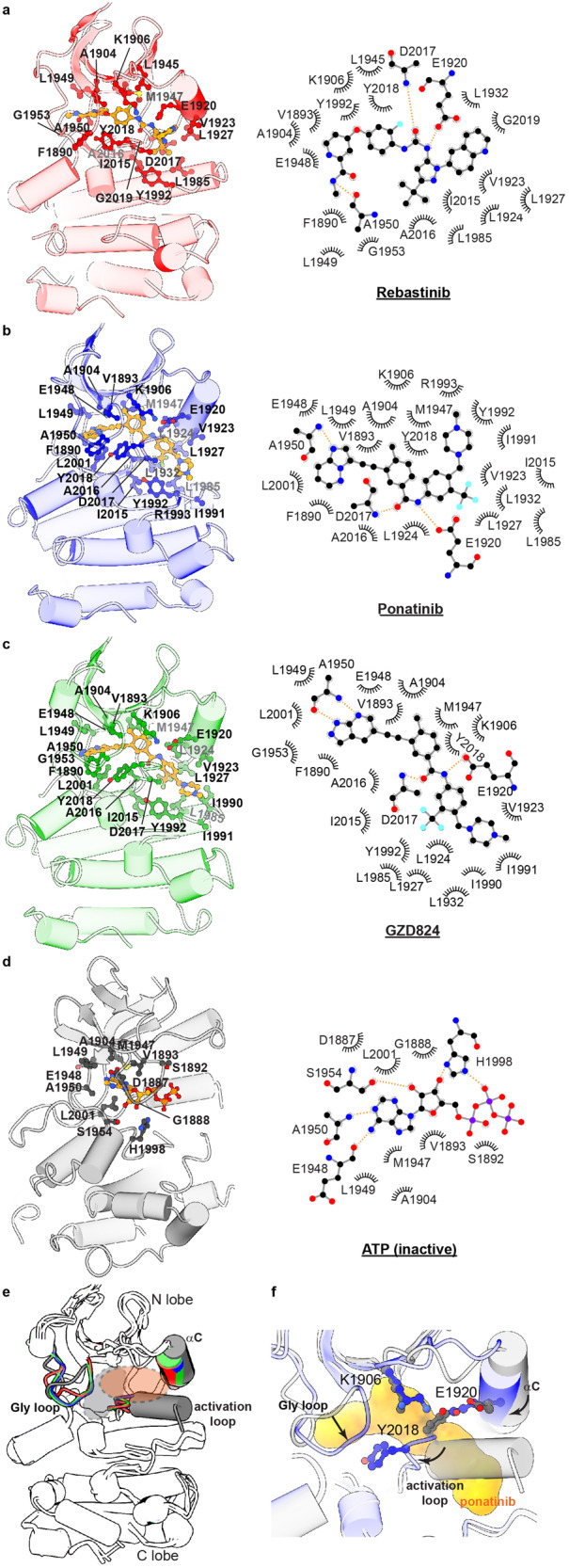
Binding of type II inhibitors to the LRRK2 KIN domain. **a**–**c** Interactions between rebastinib (**a**), ponatinib (**b**), or GZD-824 (**c**) and the LRRK2 KIN domain. Left: stereo view of the inhibitor-binding site; Right: schematic drawing of interactions formed between inhibitors and LRRK2. The bound inhibitors in yellow and surrounding residues involved in the binding are shown as ball sticks and labeled. Dashed lines indicate hydrophilic interactions. **d** Interactions between ATP and the inactive LRRK2 KIN domain (PDB 7LI4). Left: stereo view of the ATP-binding site; Right: schematic drawing of interactions formed between ATP molecule and LRRK2 KIN domain. **e** KIN domain comparison between the ATP-bound inactive LRRK2 (gray) and type-II inhibitor-bound LRRK2 structures (LRRK2–rebastinib: red, LRRK2–ponatinib: blue, LRRK2–GZD824: green). Gly loop, activation loop, and αC helix are highlighted and compared. The ATP or inhibitor binding pocket in LRRK2 is indicated by gray and orange dashed circles, respectively. **f** Comparison of the active site between ATP-bound inactive LRRK2 and the ponatinib-bound LRRK2. Differences between the Gly loop, αC helix, K1906-E1920 salt bridge, Y2018 configuration, and activation loop are indicated. Ponatinib is shown as a yellow surface.

The well-resolved KIN domains in all of these structures allow us to dissect the interactions between LRRK2 and small molecules in atomic details (Fig. 2a–d; Supplementary Fig. S5). The drug-binding poses of ponatinib and rebastinib in LRRK2 are similar to previously determined kinase–drug complexes (Supplementary Fig. S6a). GZD-824 and ponatinib are analogous compounds and bind to LRRK2 in a similar manner (Supplementary Fig. S6b). While van der Waals interactions seem to be dominant contributors, our cryo-EM structures show a recurring pattern of polar interactions between LRRK2 and type II inhibitors (Fig. 2a–c). Specifically, the main chains of A1950 and D2017 and the side chain of E1920 form polar interactions with nitrogen and oxygen atoms in all three small molecules. Notably, the αC helix that was not involved in ATP binding in the inactive state (PDB 7LHW) interacts with all three type II inhibitors (Fig. 2a–e).

**Fig. 3:**
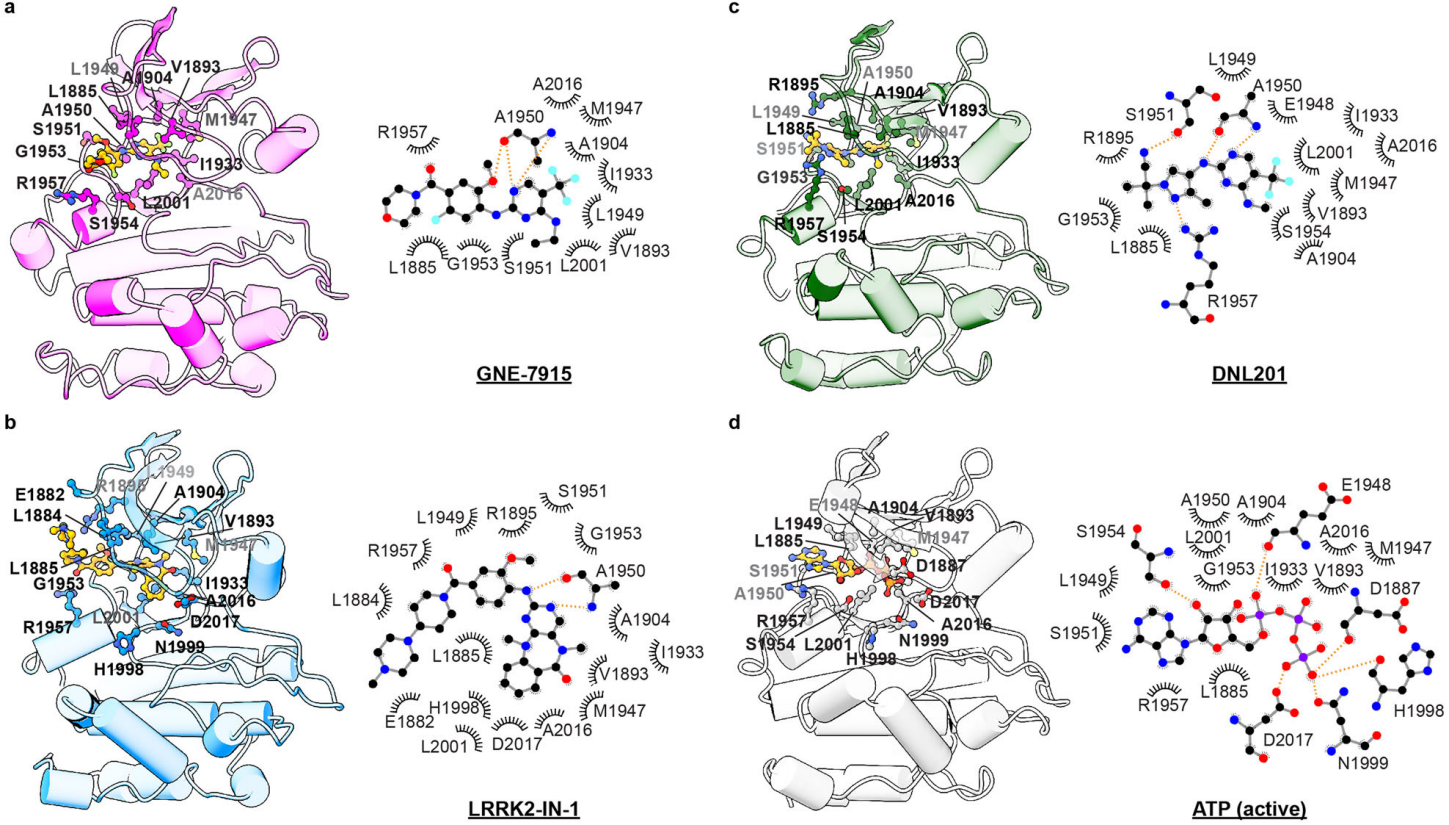
Binding of type I inhibitors to the LRRK2 KIN domain. **a**–**c** Interactions between GNE-7915 (**a**), LRRK2-IN-1 (**b**) or DNL201 (**c**) (PDB 8SMC) and LRRK2 KIN domain. Left: stereo view of the inhibitor-binding site; Right: schematic drawing of interactions formed between inhibitors and LRRK2. The inhibitors, colored in yellow, and surrounding residues are shown as ball sticks and labeled. Dashed lines indicate hydrophilic interactions. **d** Interactions between ATP and the active LRRK2 KIN domain (PDB 8FO9). Left: stereo view of the ATP-binding site; Right: schematic drawing of interactions formed between ATP molecule and the LRRK2-binding site.

Previous work reported that the LRRK2 A2016T mutation in the KIN domain does not impact kinase activity but significantly reduces its sensitivity to type II inhibitors^23^. Our structures show that Ala2016 directly interacts with all three type II inhibitors tested here, with its side chain pointing towards the inhibitors (Fig. 2a–c; Supplementary Fig. S6c), but not ATP (Fig. 3d). Introducing a hydrophilic Thr residue at this position is expected to lower the binding affinity and thus the inhibition potency of these three inhibitors.

The binding of type II inhibitors resulted in the structural re-arrangement of the ATP-binding site (Fig. 2e). The activation loop contains an alpha-helical structure (residues: D2017–C2025) in the ATP-bound inactive state (PDB 7LHW); this secondary structure “melted” and became a loop, similar to what was observed in the ATP-free structure (PDB 6VNO) (Supplementary Fig. S6d). Furthermore, rebastinib, ponatinib, and GZD-824 occupy the position that was otherwise taken by the alpha helix of the activation segment (Fig. 2f). Tyr2018 in the “DYG” motif rotates and interacts with type II inhibitors, and Lys1906 and Glu1920 form a salt bridge. Additionally, the αC helix moves towards the ATP-binding site and interacts with the small molecules (Fig. 2e, f). Since the αC helix of the KIN domain is coupled to the “Dk” helix of the COR-B domain (Supplementary Fig. S6e)^28^, we think the displacement of αC helix could contribute to the rigid-body movement of ROC-COR domains upon drug binding as shown in Supplementary Fig. S4b.

### LRRK2–type I kinase inhibitor complexes

LRRK2–GNE-7915 and LRRK2–LRRK2-IN-1 complexes share a similar overall structure with LRRK2–DNL201 (PDB 8SMC) (Supplementary Fig. S7a). We then compared the active-like type I inhibitor-bound LRRK2 structure to the active Rab29-bound LRRK2 tetramer, which contains two types of LRRK2 protomers: the periphery inactive protomer (LRRK2^peri^) and the central active protomer (LRRK2^cent^)^22^. LRRK2 with type 1 inhibitors bound show conformational differences compared to the active LRRK2^cent^ protomer in the Rab29–LRRK2 complex with a small rotational movement of ROC-COR domains (Supplementary Fig. S7b). The differences likely result from the tetramerization of LRRK2 in the Rab29–LRRK2 complex, where ROC-COR domains play a key role in mediating LRRK2 oligomerization^22^.

In our structures, type I inhibitors display well-resolved cryo-EM densities, which allow the analyses of protein–inhibitor interactions in atomic details and comparison with ATP binding in the active state (Figs. 1b, 3; Supplementary Fig. S8). Despite small differences in the overall structures, LRRK2 KIN domains with type I inhibitors and ATP are almost identical with RMSD < 0.5 Å (Supplementary Fig. S7b, c).

To understand what contributes to the selectivity of GNE-7915 and LRRK2-IN-1 for LRRK2, we compare LRRK2 and its homologous structures with type I inhibitors bound. As co-structures of protein kinases with GNE-7915 are unavailable, we performed a structural comparison between the LRRK2–LRRK2-IN-1 and humanized Roco4–LRRK2-IN-1 complexes (PDB 4YZM). LRRK2-IN-1 has an IC_50_ of 65.1 nM to LRRK2 and 1.27 mM to Roco4^31^. Previous work showed that F1107L and F1161L mutations of Roco4 lowered the IC_50_ value of LRRK2-IN-1 by more than 200 folds. Phe1107 and Phe1161 correspond to Leu1949 and Leu2001 in LRRK2, and side chains of both residues in LRRK2 point towards and interact with LRRK2-IN-1 (Supplementary Fig. S9a). Furthermore, the 1-methylpiperazine group of LRRK2-IN-1 makes more intensive interactions with the kinase N lobe of LRRK2, forming a more intensive and complementary interaction interface, which could, at least partially, contribute to another two orders of magnitude of IC_50_ difference between LRRK2 and Roco4 with F1107L and F1161L mutations (Supplementary Fig. S9b, c).

### Activation mechanism revealed by LRRK2 inhibitors

Type I and II inhibitors bind to a kinase in active-like and inactive conformations, respectively, so the LRRK2–inhibitor complexes allow us to dissect the LRRK2 activation mechanism. We use LRRK2–LRRK2-IN-1 and LRRK2–ponatinib structures to represent the type I-bound active-like and type II-bound inactive conformations in our analysis for the following reasons. First, the type I-bound LRRK2 structures are almost identical, and so are type II-bound structures (Supplementary Figs. S4b, S7a, c). Second, these two structures have slightly higher overall resolutions.

**Fig. 4:**
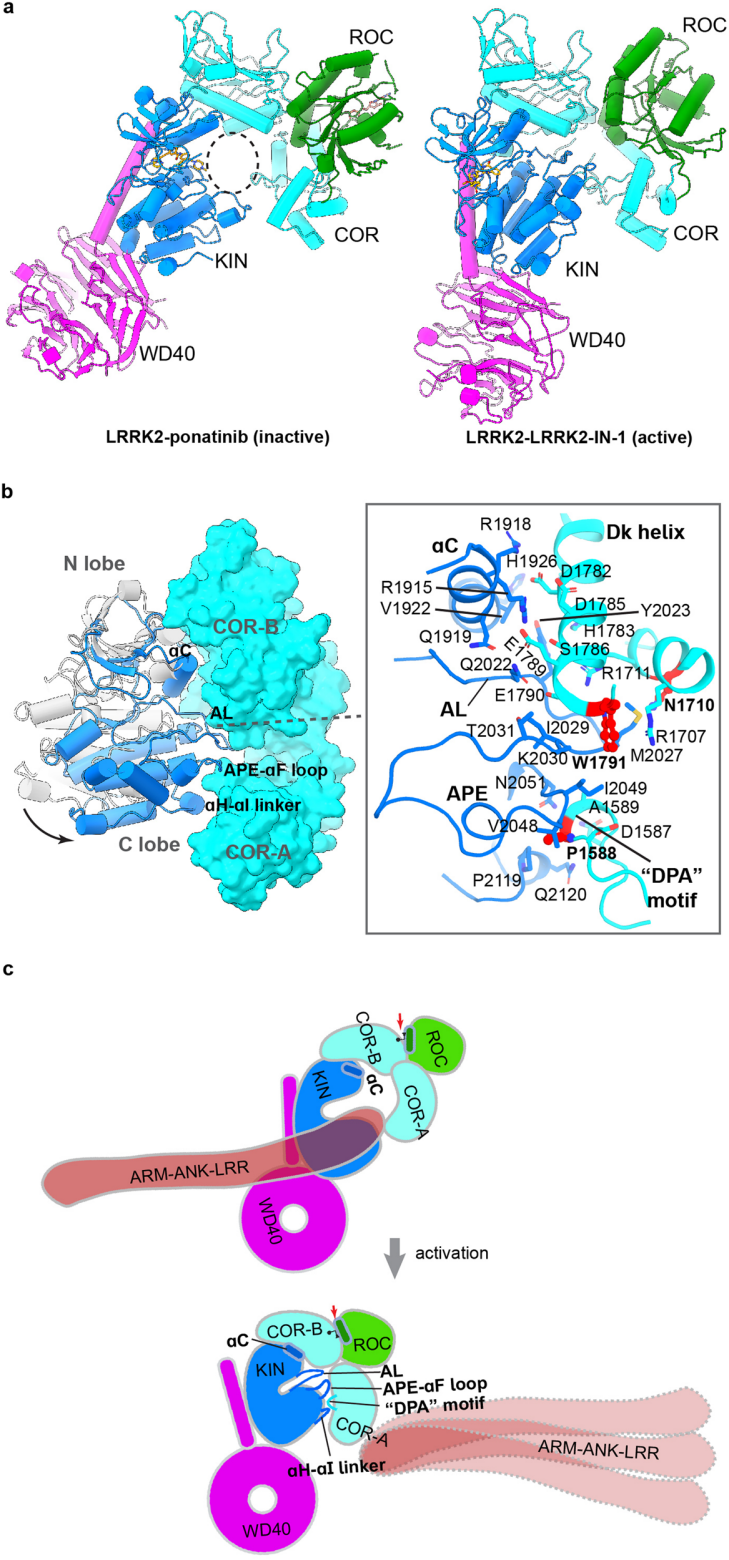
LRRK2 activation mechanism revealed by type I or type II inhibitors. **a** Structural comparison between LRRK2–ponatinib (left) and LRRK2–LRRK2-IN-1 (right) complexes. A dashed circle indicates the “central cavity” between the KIN and COR domains. **b** Movement of the KIN domain relative to the COR domain upon LRRK2-IN-1 binding compared to ponatinib binding (gray). Key structural elements are labeled. Interactions between the KIN and COR domains in the LRRK2 bound to LRRK2-IN-1 are illustrated. Sidechains of interface residues are shown. The residues, shown to be important for LRRK2 active state stabilization, are highlighted in red. **c** Cartoon representation illustrating common features in LRRK2 activation.

There are significant conformational differences between the active-like LRRK2–LRRK2-IN-1 and the inactive LRRK2–ponatinib complex structures in the ROC-COR-KIN-WD40 domains (Fig. 4). The cavity shaped by KIN and COR domains in the active-like LRRK2–LRRK2-IN-1 complex is sealed by KIN–COR interactions (Fig. 4a, b). This is accompanied with the opening of COR-A and COR-B subdomains (Supplementary Fig. S10a), more intensive interactions between the KIN N lobe and COR-B, and a novel interface between the KIN C lobe and COR domain (Supplementary Fig. S10b). The new KIN–COR interface is almost identical to what was observed in the LRRK2^cent^ of the Rab29–LRRK2 complex despite a small displacement of the COR-A domain caused by protein oligomerization (Supplementary Fig. S9c)^22^. Specifically, the αC helix, activation loop, APE-αF loop, and αH-αI linker of the KIN domain interact with the COR-B and a “DPA” motif (D1587–A1589) of the COR-A domain (Fig. 4b; Supplementary Fig. S10b, c). Key residues like Trp1791, Asn1710, and Pro1588, shown to play a critical role in LRRK2 activation^22^, contribute to the KIN–COR interaction (Fig. 4b). These observations suggest that LRRK2 activation by inhibitors and Rab29 has common key structural features — COR-A and COR-B open up and allow the docking of the closed KIN domain to stabilize the active conformation^22,28^.

The “seesaw” motion of the ROC αC helix was also observed in the small-molecule-mediated activation, with Tyr1699 being the pivot point. The side chain of Tyr1699 flips during the inactive-to-active transition (Supplementary Fig. S10d). The ROC αC helices are similar in the inactive states but differ in the active LRRK2-IN-1-bound and LRRK2^cent^ structures. In LRRK2–LRRK2-IN-1, the ROC αC helix shows a smaller movement (Supplementary Fig. S10e). We thus believe that the “seesaw” motion and flipping of Tyr1699 are also key features of kinase activation, though the degree of “seesaw” motion could vary among different activation mechanisms.

We summarized common features of LRRK2 activation in Fig. 4c. First, N-terminal ARM-ANK-LRR domains manifest conformational changes, eliminating the shielding effect of the LRR domain on the kinase active site. Second, the COR domain opens up to accommodate the closed kinase domain — the αC helix of KIN tilts towards the COR-B domain; the activation loop and APE-αF loop insert into the cleft between COR-A and COR-B; and the APE-αF loop and the αH-αI linker grip the “DPA” motif of the COR-A domain. Meanwhile, the WD40 domain rotates to cope with the KIN domain movement. Third, the αC helix of the ROC domain undergoes a “seesaw” motion on COR-B using the Tyr1699 as the pivot point, and the side chain of Tyr1699 flips upon activation.

### Molecular dynamics (MD) simulation of LRRK2 KIN domain

**Fig. 5:**
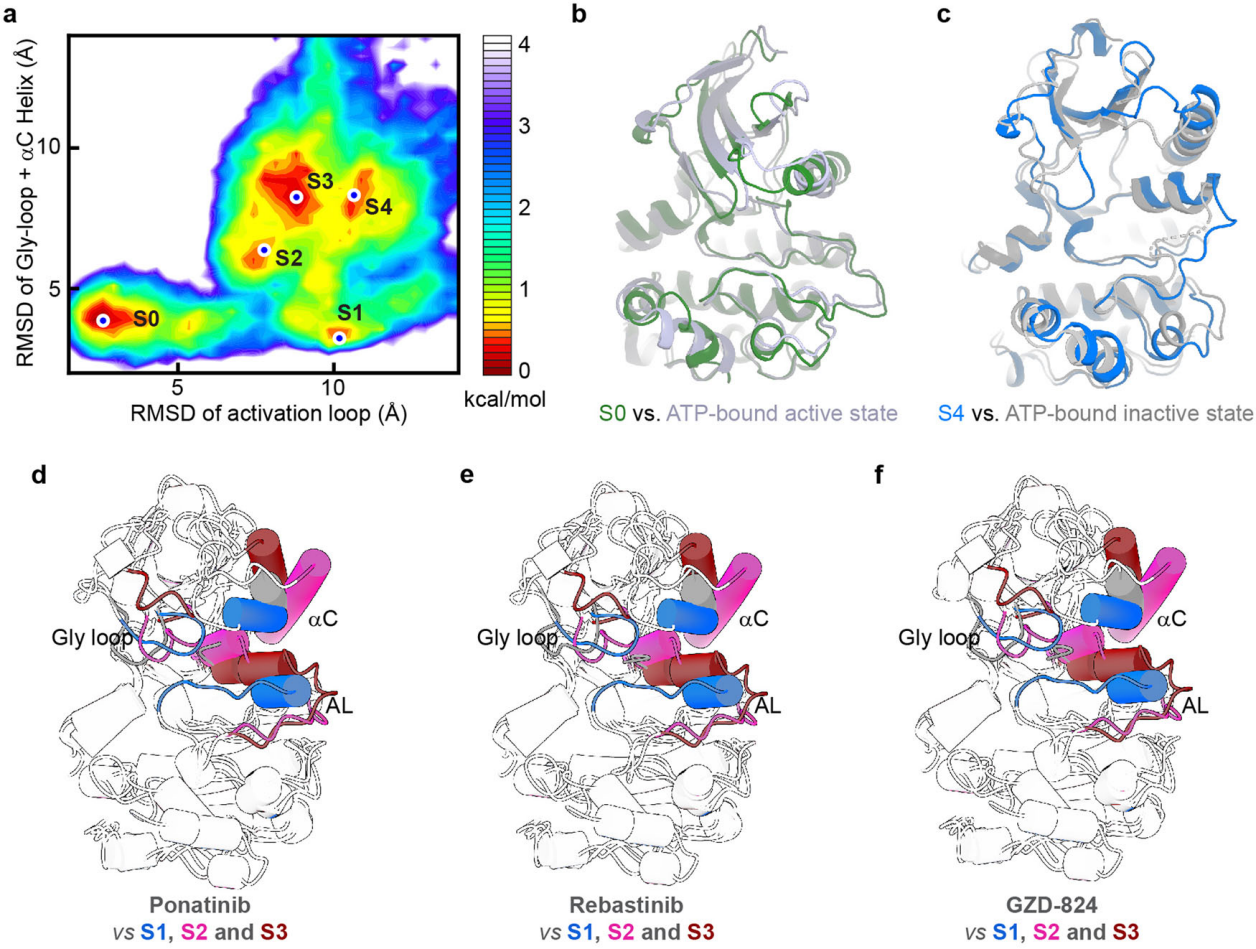
MD simulation of LRRK2 KIN domain. **a** Metastable states (S0–S4) revealed by GaMD simulations. The free energy profile was projected along two RMSD coordinates (see the section “Materials and methods”). **b** Comparison between the S0 state and the ATP-bound active state. **c** Comparison between the S4 state and the ATP-bound inactive state. **d** LRRK2–ponatinib vs S1–S3 states. **e** LRRK2–rebastinib vs S1–S3 states. **f** LRRK2–GZD-824 vs S1–S3 states. Key structural elements, including Gly loop, activation loop (AL), and αC helix, are highlighted with S1–S3 in blue, magenta, and brown, respectively.

Using cryo-EM, we and others have captured several conformations of the LRRK2 KIN domain, including ATP-bound active and inactive states, drug-bound active-like and inactive states, and the ATP-free inactive state^21,22,30,32^. These structural states, with biophysical characterizations^28,29,33,34^, provided valuable frameworks for structure-based drug discovery using computational screening and docking methods. We ask if there are other stable states in the conformational landscape of the LRRK2 KIN domain that we can explore for drug discovery. Here, we combined AlphaFold2 modeling^35^ and Gaussian accelerated MD (GaMD) simulations^36,37^ to effectively sample the conformational space for the apo KIN domain (residues 1860–2138). The results revealed five metastable states (S0–S4), as illustrated by the 2D free energy profile shown in Fig. 5a. The S0 state has features of an active kinase and resembles the conformation of the ATP-bound active state (Fig. 5b), and the S4 state is most close to the ATP-bound inactive state (Fig. 5c). States S1, S2, and S3 lie between S0 and S4 and represent intermediate states between active and inactive conformations.

Based on the energy landscape, multiple pathways exist for the transition from an inactive to an active KIN domain. For example, in the pathway S4 → S1 → S0, the major αC helix movement towards the ligand binding site happens first, subsequently followed by the melting and extension of the alpha-helical conformation of the activation loop. Alternatively, in the pathway, S4 → S3 → S2 → S0, the major movement of the αC helix takes place only after partial melting and extension of the activation loop (Supplementary Video S1). The binding pocket configurations observed in these intermediate states could serve as potential targets for in-silico small-molecule screening. Complemented by further experimental validations of computational predictions, our approach should offer new opportunities for LRRK2 inhibitor development. On the other hand, the LRRK2–type II inhibitor complexes show significant differences with S1–S3 states (Fig. 5d–f), indicating that the induced-fit theory holds for LRRK2 inhibition by small molecules. As such, high-throughput screen assays like what we used in Supplementary Fig. S1a could provide novel initial leads that are otherwise difficult to be gained by computational docking.

## Discussion

This study reports experimental workflows for determining LRRK2–inhibitor complex structures to facilitate structure-based drug discovery. LRRK2 is one of the most promising drug targets for PD, and numerous pharmaceutical companies are actively pursuing LRRK2-selective kinase inhibitors as potential PD therapeutic tools. Our experimental approaches for LRRK2–inhibitor structural determination will allow validation, characterization, and optimization of ATP-competitive lead compounds, including type I, type II, and LRRK2 G2019S selective inhibitors^18,23,38,39^, during clinical trials.

The structures of LRRK2–inhibitor complexes serve as the structural templates for the structure-guided design of LRRK2-specific inhibitors. Due to the potential concerns of type I inhibitors, which cause LRRK2 filamentation, LRRK2-selective type II inhibitors are actively pursued. The LRRK2–GZD824, LRRK2–rebastinib, and LRRK2–ponatinib complexes provide structural templates for designing LRRK2-specific type II inhibitors. Additionally, we notice that the activation loop of LRRK2 forms a unique helical structure, which “melts” upon activation or type II inhibitor binding. We speculate that it would be possible to take advantage of this unique structural feature to achieve type II inhibitor specificity. Additionally, the most common LRRK2 PD mutation, the G2019S mutation, also adopts the same conformation in the inactive conformation^21^.

Our study, along with other published work, reveals key structural features during the inactive-to-active transition of LRRK2 (Fig. 4; Supplementary Fig. S10). These observations, such as the stabilization of the activation loop, crosstalk between the Dk helix of the COR-B domain and αC helix of the KIN domain, and the biochemical flexibility of the N-terminal domains, are consistent with the previous structural studies and hydrogen–deuterium exchange mass spectrometry (HDX-MS) data with MD simulation analysis^28,29,33,34^.

Lastly, our MD simulation analysis indicates the existence of metastable states that we could uncover by experimental methods like NMR and use for drug discovery by computational docking endeavors. On the other hand, we believe that high-throughput assays have the potential to discover novel leads that can stabilize the LRRK2 KIN domain in conformations of low occupancies. Thus, we could maximize the LRRK2 drug discovery by computational and experimental screens.

## Materials and methods

### Cell lines

Sf9 cells were cultured in Sf-900 III SFM medium (GIBCO) at 27 °C. HEK293F cells were cultured in Freestyle 293 medium (GIBCO) supplemented with 2% FBS (GIBCO) and 1% Pen/Strep at 37 °C.

### Cloning, expression, and purification of human LRRK2

The full-length LRRK2 construct was expressed and purified as described^21,22^. The LRRK2^RCKW^ construct was subcloned from the pDEST53-LRRK2-WT vector (Addgene: 25044), and the M1732R mutation was introduced for structural and biochemical studies using the QuickChange Site-Directed Mutagenesis kit (Strategene) to prevent protein aggregation^21^. A GFP tag followed by a preScission protease cleavage site was engineered at the N terminus of full-length LRRK2 or LRRK2^RCKWm^, which was cloned into the BacMam expression vector^40^. Bacmids carrying both LRRK2 constructs were generated in *E. coli* DH10Bac cells (Invitrogen). Recombinant baculoviruses were produced and amplified in Sf9 cells. Proteins were expressed in HEK293F cells infected with 10% baculovirus at a density of ~2–3 × 10^6^ cells/mL. Infected cells were incubated at 37 °C overnight, and protein expression was induced by adding 10 mM sodium butyrate. Cells were cultured at 30 °C for another 48–60 h before harvest.

For full-length LRRK2 purification, the cell pellet was resuspended in lysis buffer (20 mM Tris pH 8.0, 200 mM NaCl, 5% glycerol, 2 mM DTT, and protease inhibitors), and then cells were lysed by brief sonication. LRRK2 was separated from the insoluble fraction by high-speed centrifugation (38,000× *g* for 1 h), and incubated with CNBr-activated sepharose beads (GE Healthcare) coupled with high-affinity GFP nanobodies (GFP-NB)^41^. The GFP tag was cleaved by preScission protease at 4 °C, and LRRK2 was further purified by size-exclusion chromatography with a Superose 6 Increase 10/300 GL column (GE Healthcare) equilibrated with 20 mM Tris pH 8.0, 200 mM NaCl, and 2 mM DTT. The purified protein was collected and concentrated to 12 mg/mL (OD_280_) using a 100-kDa MWCO centrifugal device (Ambion), flash-frozen in liquid nitrogen, and stored at −80 °C.

For LRRK2^RCKWm^ purification, the cell pellet was resuspended in lysis buffer (20 mM HEPES pH 7.4, 300 mM NaCl, 5% glycerol, 2 mM DTT and protease inhibitors), and then cells were lysed by brief sonication. LRRK2^RCKWm^ was separated from the insoluble fraction by high-speed centrifugation (38,000× *g* for 1 h) and incubated with CNBr-activated sepharose beads (GE Healthcare) coupled with high-affinity GFP nanobodies (GFP-NB)^41^. The beads were washed with lysis buffer plus 1 mM MgCl_2_ and 2 mM ATP to remove contamination of heat shock proteins. The GFP tag was cleaved by preScission protease at 4 °C, and LRRK2^RCKWm^ was further purified by size-exclusion chromatography with a Superose 6 Increase 10/300 GL column (GE Healthcare) equilibrated with 20 mM HEPES pH 7.4, 200 mM NaCl and 2 mM DTT. The purified protein was collected and concentrated to 2.5 mg/mL (OD_280_) using a 100-kDa MWCO centrifugal device (Ambion), flash-frozen in liquid nitrogen, and stored at –80 °C.

### Measurement of LRRK2 inhibition in vitro

The LRRK2 inhibition assay was performed using a commercially available ADP-Glo^TM^ Kinase Assay kit (Promega). Dilutions of inhibitor were prepared by serial dilution of a 50–100 mM stock solution of inhibitor in DMSO into ddH_2_O. The standard LRRK2 kinase reaction solution (5 μL) consisted of 40 mM Tris–HCl pH7.4, 20 mM MgCl_2_, 0.1 mg/mL BSA, 2 mM DTT, 10 μM ATP, 190 μM LRRKtide, and varying concentrations of the inhibitor, and the reaction was initiated by the addition of 15 nM LRRK2^RCKWm^ enzyme followed by incubation at room temperature for 100 min. Then 5 μL ADP-Glo^TM^ reagent was added to the mixture to stop the kinase reaction and deplete unreacted ATP by further incubation at room temperature for 45 min. 10 μL of Kinase Detection reagent was added to convert ADP to ATP and introduce luciferase and luciferin to detect ATP by incubating at room temperature for 30 min. The luminescence signal was recorded at 520 nm with an integration time of 1 s in an Nunc 384-well plate using a POLARstar Omega microplate reader. The percentage of kinase activity compared to control without inhibitor was plotted against the log concentration of the inhibitor, and the data were fitted to a four-parameter logistic curve in GraphPad Prism version 8 (GraphPad, San Diego, CA). All experiments were performed with at least three repeats, and the data were presented as mean ± SD.

### Cryo-EM sample preparation

Cryo-EM grids were prepared with a Vitrobot Mark IV (FEI). Quantifoil R1.2/1.3 300 Au holey carbon grids (Quantifoil) were glow-discharged for 30 s. For cryo-EM sample preparation of LRRK2 in the presence of type II inhibitors, the purified full-length LRRK2 protein was incubated with 200 μM GZD-824, ponatinib or 100 μM rebastinib on ice for 30 min and prior to vitrification centrifuged at 4 °C (38,000× *g* for 10 min) to remove potential precipitation. 2.3 mM fluorinated Fos-Choline-8 was added right before freezing the grids, followed by a single application of 3.5 μL of protein sample onto the grids with a 3.5-s blot time, blot force of –3 under 16 °C and 95% relative humidity and plunge-frozen in liquid nitrogen-cooled liquid ethane. For cryo-EM sample preparation of LRRK2 in the presence of type I inhibitors, the purified LRRK2^RCKWm^ protein was incubated with 100 μM LRRK2-IN-1 or GNE-7915 on ice for 30 min and prior to vitrification centrifuged at 4 °C (38,000× *g* for 10 min) to remove potential precipitation. 2.3 mM fluorinated Fos-Choline-8 was added right before freezing the grids, followed by a double application of 3.0 μL of protein sample onto the grids with a manual first blotting step, with a 3.5-s blot time, blot force of –3 under 16 °C and 95% relative humidity after second sample loading and plunge-frozen in liquid nitrogen-cooled liquid ethane.

### Cryo-EM data acquisition and processing for LRRK2–GZD-824 complex

The LRRK2–GZD-824 dataset was collected on a Titan Krios (Thermo Fisher Scientific) transmission electron microscope equipped with a K3 direct electron detector and post-column GIF energy filter (Gatan). Data collection was performed in an automated manner using EPU (Thermo Fisher Scientific). Movies were recorded at defocus values from –0.6 to –1.8 μm at a magnification of 81kx in super-resolution mode, corresponding to a pixel size of 0.53 Å. During 4.0 s exposure, 60 frames were collected with a total electron dose of ~67 e^–^/Å^–2^ (at a dose rate of 1.1 e^–^/frame/Å^2^). In total, 9528 images were collected. Motion correction was performed on raw super-resolution movie stacks and binned by 2 using MotionCor2^42^. Contrast transfer function (CTF) estimation was performed using Gctf^43^. Prior to particle picking, micrographs were analyzed for good power spectrum, and the bad ones were discarded (with 9413 good images remaining).

Particles were selected using the template picker with reported inactive full-length LRRK2 structures^21^ as a reference in cryoSPARC^44^ and extracted using a binning factor of 2. Several rounds of the 2D classification were performed to eliminate ice artifacts, carbon edges, and false-positive particles containing noise. During 2D classification, two groups of good classes were observed, corresponding to the LRRK2 monomer and dimer states, respectively. Both groups were selected, and ab initio reconstruction was performed. In order to further separate the LRRK2 monomer and dimer states, we performed Heterogeneous refinement in cryoSPARC. As a result, 69,779 particles were assigned to the monomer class and 78,975 particles to the dimer. Both 3D classes were further refined using cryoSPARC after extraction of unbinned particles corresponding to each identified sub-set. For the LRRK2 monomer state, we performed a standard NU-refinement without imposing symmetry. For the LRRK2 dimer state, we performed NU-refinement by applying C2 symmetry, and then symmetry expansion followed by focused refinement to further improve the resolution of each LRRK2 protomer without N-terminal ARM domain. All resolution estimates were calculated according to the gold-standard Fourier shell correlation (FSC) using the 0.143 criterion^45^. Local resolution was estimated in cryoSPARC. The density maps were *B*-factor sharpened in cryoSPARC and used to produce figures and build models.

### Cryo-EM data acquisition and processing for LRRK2–ponatinib complex

The LRRK2–ponatinib dataset was collected similarly, except for the following differences. Movies were recorded at defocus values from –0.6 to –1.8 μm at a magnification of 130 kx in hardware binning mode, corresponding to a pixel size of 0.6485 Å at the specimen. During 2.0 s exposure, 60 frames were collected with a total electron dose of ~68 e^–^/Å^–2^ (at a dose rate of 1.1 e^–^/frame/Å^2^). In total, 27,611 images were collected. Motion correction was performed on hardware-binned movie stacks and binned by 1 using MotionCor2^42^. CTF estimation was performed using Gctf^43^. After the selection of high-quality micrographs, 24,254 images were used during the data process.

The LRRK2–ponatinib dataset was processed similarly in cryoSPARC. Briefly, particles were selected using the template picker and extracted using a binning factor of 4. Several rounds of the 2D classification were performed and two groups of good classes were observed, corresponding to the LRRK2 monomer and dimer states, respectively. Both groups were selected, and ab initio reconstruction was performed. Heterogeneous refinement was performed to further separate the LRRK2 monomer and dimer states. As a result, 95,805 particles were assigned to the monomer class and 75,849 particles to the dimer. Both 3D classes were further refined after extraction of unbinned particles corresponding to each identified sub-set. For the LRRK2 monomer state, we performed a standard NU-refinement without imposing symmetry. For the LRRK2 dimer state, we performed NU-refinement by applying C2 symmetry, and then symmetry expansion followed by focused refinement to further improve the resolution of each LRRK2 protomer without N-terminal ARM domain. All resolution estimates were calculated according to the gold-standard FSC using the 0.143 criterion^45^. Local resolution was estimated in cryoSPARC. The density maps were *B*-factor sharpened in cryoSPARC and used to produce figures and build models.

### Cryo-EM data acquisition and processing for LRRK2–rebastinib complex

The LRRK2–rebastinib dataset was collected similarly, except for the following differences. Movies were recorded at defocus values from –0.6 to –1.8 μm at a magnification of 130 kx in hardware binning mode, corresponding to a pixel size of 0.6485 Å at the specimen. During 2.0 s exposure, 70 frames were collected with a total electron dose of ~68 e^–^/Å^–2^ (at a dose rate of 1.0 e^–^/frame/Å^2^). In total, 22,359 images were collected. Motion correction was performed on hardware-binned movie stacks and binned by 1 using MotionCor2^42^. CTF estimation was performed using Gctf^43^. After the selection of high-quality micrographs, 22,102 images were used during the data process.

The LRRK2–rebastinib dataset was processed similarly in cryoSPARC. Briefly, particles were selected using the template picker and extracted using a binning factor of 4. Several rounds of the 2D classification were performed and only one group of good classes was observed, corresponding to the LRRK2 dimer state. Good 2D classes were selected, and ab initio reconstruction was performed. Heterogeneous refinement was performed to further separate the LRRK2 dimer state from bad classes. As a result, 75,005 good particles were selected. The 3D class was further refined after the extraction of unbinned particles corresponding to the identified sub-set. We performed NU-refinement by applying C2 symmetry, and then symmetry expansion followed by focused refinement to further improve the resolution of each LRRK2 protomer without N-terminal ARM domain. All resolution estimates were calculated according to the gold-standard FSC using the 0.143 criterion^45^. Local resolution was estimated in cryoSPARC. The density map was *B*-factor sharpened in cryoSPARC and used to produce figures and build models.

### Cryo-EM data acquisition and processing for LRRK2^RCKWm^ bound to LRRK2-IN-1 or GNE-7915

The LRRK2^RCKWm^–LRRK2-IN-1 dataset was collected similarly, except for the following differences. Movies were recorded at defocus values from –0.6 to –2.0 μm at a magnification of 130 kx in hardware binning mode, corresponding to a pixel size of 0.6485 Å at the specimen. During 2.0 s exposure, 70 frames were collected with a total electron dose of ~63 e^–^Å^–2^ (at a dose rate of 0.9 e^–^/frame/Å^2^). For the LRRK2^RCKWm^–GNE-7915 dataset, movies were recorded at defocus values from –0.8 to –2.4 μm at a magnification of 130 kx in hardware binning mode, corresponding to a pixel size of 0.6485 Å at the specimen. During 2.0 s exposure, 70 frames were collected with a total electron dose of ~62 e^–^/Å^–2^ (at a dose rate of 0.9 e^−^/frame/Å^2^). In total, 37,081 and 28,882 images were collected for the LRRK2^RCKWm^–LRRK2-IN-1 and LRRK2^RCKWm^–GNE-7915 datasets, respectively. Motion correction was performed on hardware-binned movie stacks and binned by 1 using MotionCor2^42^. CTF estimation was performed using Gctf^43^. After the selection of high-quality micrographs, 36,884 and 28,530 images were used during further data process for the LRRK2^RCKWm^–LRRK2-IN-1 and LRRK2^RCKWm^–GNE-7915 datasets, respectively.

Particles were first selected using the Blob picker in cryoSPARC^44^ and extracted using an initial binning factor of ~2.5. Several rounds of 2D classification were performed to eliminate ice artifacts, carbon edges, and false-positive particles containing noise. Potential good 2D classes were selected and ab initio reconstruction was performed. One good 3D class was obtained, corresponding to an active-like LRRK2 state. All generated initial 3D classes and selected particles after 2D classification were used as input and rounds of Heterogeneous refinement were performed to further sort the dataset into a more homogeneous subset. The 3D class was further refined after the extraction of unbinned particles corresponding to the identified sub-set. We performed a standard NU refinement without imposing symmetry. The best class contained 187,407 and 95,644 particles and yielded 3.5 and 3.8 Å overall resolution maps for the LRRK2^RCKWm^–LRRK2-IN-1 and LRRK2^RCKWm^–GNE-7915 structures, respectively. All resolution estimates were calculated according to the gold-standard FSC using the 0.143 criteria^45^. Local resolution was estimated in cryoSPARC. The density map was *B*-factor sharpened in cryoSPARC and used to produce figures and build models.

### Model building and refinement

All structural models were built using the highest-resolution maps obtained for each complex. The reported structures of LRRK2 (PDB 7LI4)^21^ and the central LRRK2 molecule of the Rab29-LRRK2 tetramer state (PDB 8FO9)^22^ were used as starting points for the LRRK2–type II and LRRK2^RCKWm^–type I inhibitors, respectively. Protein models were fitted and adjusted into the cryo-EM maps using Chimera^46^ and Coot^47^. Inhibitor model was generated via elBOW software available in Phenix package^48^ with SMILES file of the molecule as input and was further fitted and adjusted into the cryo-EM map and incorporated into the structure model. The structural model was refined against the map using the real space refinement module with secondary structure and non-crystallographic symmetry restraints in the Phenix package^48^. FSC curves were calculated between the refined model and the full map. The geometries of the models were validated using MolProbity^49^. All the figures were prepared in PyMOL (Schrödinger, LLC.), UCSF Chimera^46^ and UCSF ChimeraX^50^.

### AlphaFold2 modeling and GaMD simulations of the KIN domain

AlphaFold2^35^ was used to prepare an initial conformational ensemble, which served as starting configurations for MD simulations. Specifically, the ColabFold package^51^ was used to facilitate sampling of the structural heterogeneity through template-based predictions. Each inhibitor-bound structure presented in this study was used as a single template input for ColabFold. Additional templates also included the LRRK2 KIN domain structures of the ATP-bound active LRRK2, inactive LRRK2 (PDB 7LHW), and ATP-free LRRK2 (PDB 6VNO). For each template, 20 independent prediction runs were launched with random seeds, while setting msa mode to single_sequence and the number of recycles to 1. The resulting models with an average predicted local distance difference test (pLDDT)^35^ score above 75 were chosen for clustering analysis. The centroid structures of 23 top clusters were selected for subsequent MD simulations.

Each selected structure was solvated in a water box with 150 mM NaCl. All the MD simulations were performed using the GPU version of Amber18^52^ with the ff14SB force field^53^. Firstly energy minimization was carried out while applying harmonic restraints to protein atoms with a spring constant of 1 kcal/(mol Å ^2^). With the same positional constraints, a following 5 ns equilibration simulation was performed at 300 K, which was maintained by Langevin dynamics with a friction coefficient of 1 ps^−1^. Particle Mesh Ewald^54^ was used for full-system periodic electrostatics and a 9 Å cutoff was applied to Lennard–Jones interactions. Then GaMD was employed to enhance the sampling of protein conformational transitions^36^. Here the GaMD protocol included a 10-ns conventional MD stage to gather statistics for calculating initial GaMD acceleration parameters, followed by a 290-ns GaMD stage. During the GaMD stage, both total potential energy boost and dihedral energy boost were applied to the system, each having a 4 kcal/mol upper limit of the standard deviation for accurate reweighting. The GaMD acceleration parameters were updated adaptively during the first 100 ns. The accumulated GaMD trajectories amounted to 6.67 µs. The two-dimensional free energy profile was obtained via the reweighting approach described in ref. ^37^. For the RMSD calculations, the reference frame was the ATP-bound active state, and all the frames were aligned to the core helices of the KIN-domain C-lobe.

### Supplementary information

Supplementary Figures and Tables

Supplementary Movie S1

## Data Availability

The cryo-EM maps of the LRRK2–inhibitor complexes have been deposited in the Electron Microscopy Data Bank under the accession codes EMD-42020 (LRRK2–rebastinib), EMD-42019 (LRRK2–ponatinib), EMD-41985 (LRRK2–GZD-824), EMD-41982 (LRRK2^RCKWm^–GNE-7915) and EMD-29340 (LRRK2^RCKWm^–LRRK2-IN-1). The corresponding coordinates have been deposited in the Protein Data Bank under the accession codes 8U8B (LRRK2–rebastinib), 8U8A (LRRK2–ponatinib), 8U7L (LRRK2–GZD-824), 8U7H (LRRK2^RCKWm^–GNE-7915) and 8FO7 (LRRK2^RCKWm^–LRRK2-IN-1).
