# Author Correction: Pharmacology of LRRK2 with type I and II kinase inhibitors revealed by cryo-EM

**DOI:** 10.1038/s41421-024-00660-5

**Published:** 2024-02-26

**Authors:** Hanwen Zhu, Patricia Hixson, Wen Ma, Ji Sun

**Affiliations:** 1 Department of Structural Biology, St. Jude Children’s Research Hospital, Memphis, TN USA; 2 Department of Physics, University of Vermont, Burlington, VT USA

**Keywords:** Structural biology, Post-translational modifications

Correction to: *Cell Discovery*

10.1038/s41421-023-00639-8 published online 23 January 2024

As per Mansoore Esmaili’s request, we would like to remove “M. Esmaili for help with kinase inhibition assay” from the Acknowledgements section. We apologize for the inconvenience.

