# Supplementary material for: Pharmacology of LRRK2 with type I and II kinase inhibitors revealed by cryo-EM
Source: Cell Discov. 2024 Jan 23;10:10. doi: 10.1038/s41421-023-00639-8 (PMC10805800; doi:10.1038/s41421-023-00639-8)
Supplement: Supplementary file 1 — Supplementary Figures and Tables [file 41421_2023_639_MOESM1_ESM.pdf]

Supplementary Information for

Pharmacology of LRRK2 with type I and II kinase inhibitors revealed by cryo-EM

Hanwen Zhu[1], Patricia Hixson[1], Wen Ma[2*] and Ji Sun[1*]

[1] Department of Structural Biology, St. Jude Children's Research Hospital, Memphis, TN, USA

[2] Department of Physics, University of Vermont

* Corresponding authors: wen.ma@uvm.edu (Wen Ma) and ji.sun@stjude.org (Ji Sun)

This file includes:

Supplementary Figures S1 to S10

Supplementary Tables S1 to S2

Supplementary Video S1 title

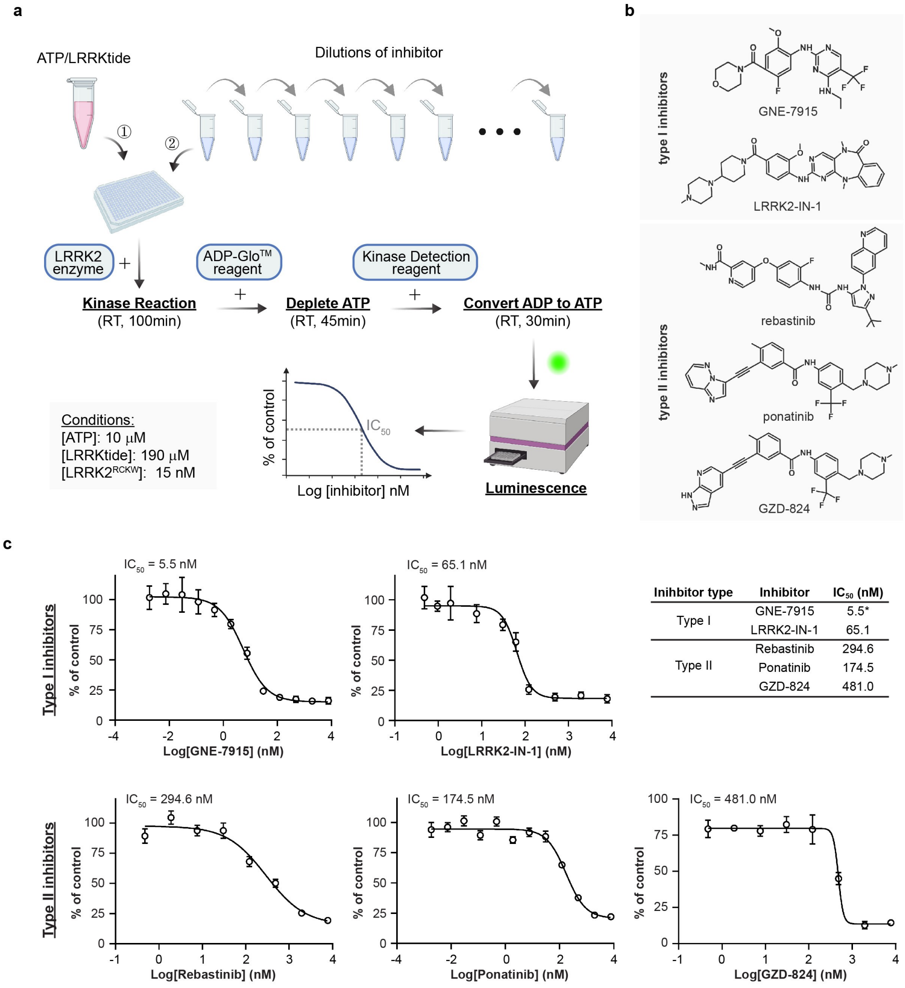

Supplementary Fig. S1 Biochemical characterization of LRRK2 and its selective inhibitors. a The ADP-Glo assay to test the inhibition of LRRK2 by small molecules. **b** Chemical structures of the representative LRRK2 kinase type I and type II inhibitors. **c** Measurement of LRRK2 kinase inhibition by type I and type II inhibitors. The inhibitors prevented LRRK2 phosphorylation of LRRKtide substrate with nanomolar IC_{50} values. Note (*): The IC_{50} of GNE-7915 (5.5 nM) is close to ½ enzyme concentration (15 nM/2=7.5 nM), suggesting the measured IC_{50} is not accurate under our experimental settings and the real IC_{50} is likely much smaller. However, we could only achieve a good signal/noise ratio when the enzyme concentration is higher than 5 nM with some inhibitors. In this study, we performed all measurements with $LRRK2^{RCKWm}$ at a concentration of 15 nM, which provides a robust measurement for all inhibitors.

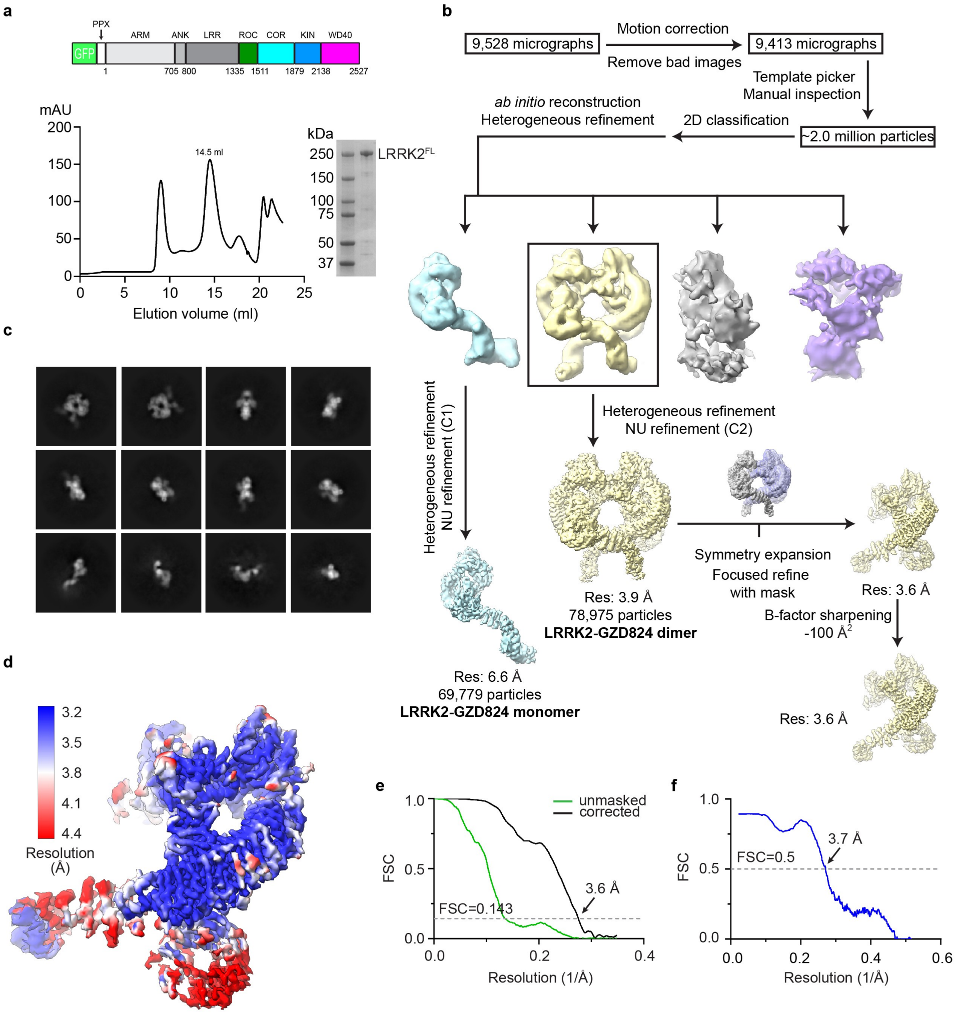

Supplementary Fig. S2 Cryo-EM analysis of the LRRK2-GZD-824 complex. a Scheme of mammalian expression construct (top) and size-exclusion chromatography (SEC) purification profile (bottom) of the human full-length LRRK2 used for structural analysis of LRRK2-type II inhibitors. **b** A simplified flow chart of cryo-EM data processing. **c** Representative 2D classes of the LRRK2-GZD-824 complex from cryoSPARC. **d** Local resolution of the LRRK2-GZD-824 complex. Inset: zoom-in view of the inhibitor binding site. **e** Fourier Shell Correlation (FSC) curves for the overall resolution of the LRRK2-GZD-824 complex. **f** FSC curve of map-model fitting.

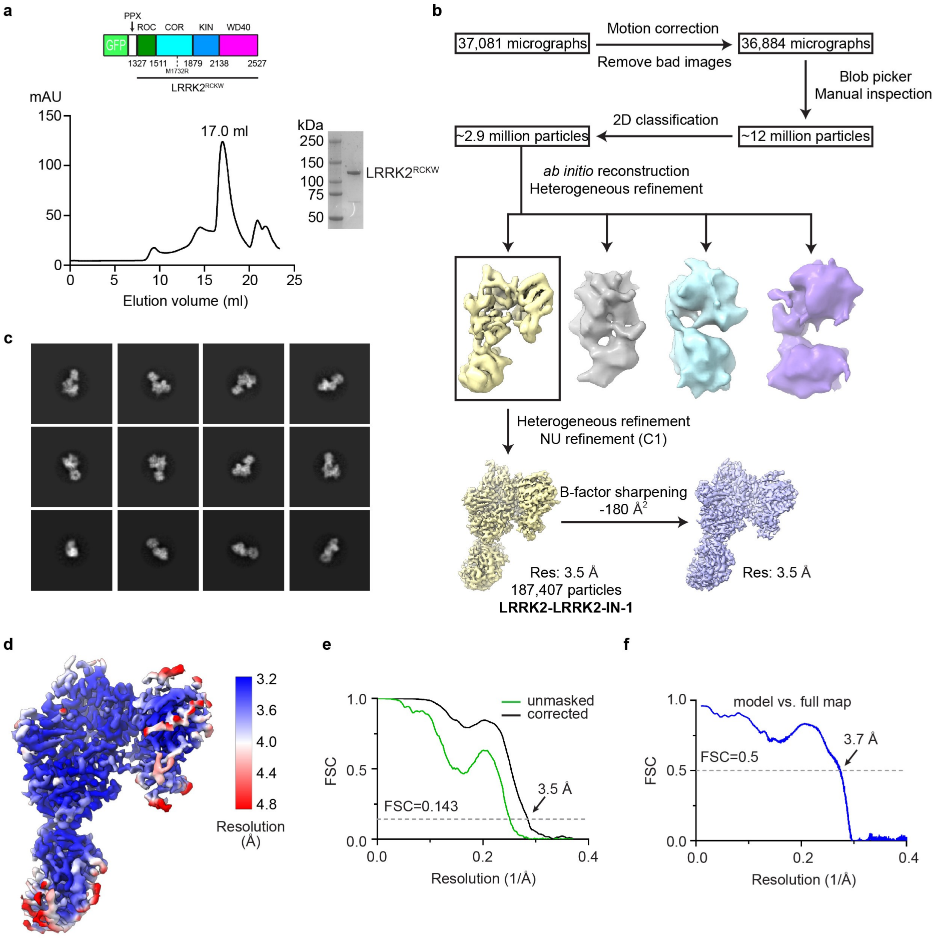

Supplementary Fig. S3 Cryo-EM analysis of the LRRK2-LRRK2-IN-1 complex. a Scheme of mammalian expression construct (top) and size-exclusion chromatography (SEC) purification profile (bottom) for LRRK2^{RCKWm} used for cryo-EM study of LRRK2 in complex with type-I inhibitors. **b** A simplified flow chart of cryo-EM data processing. **c** Representative 2D classes of the LRRK2-LRRK2-IN-1 complex from cryoSPARC. **d** Local resolution of the LRRK2-LRRK2-IN-1 complex. **e** Fourier Shell Correlation (FSC) curves for the overall resolution of the LRRK2-LRRK2-IN-1 complex. **f** FSC curve of map-model fitting.

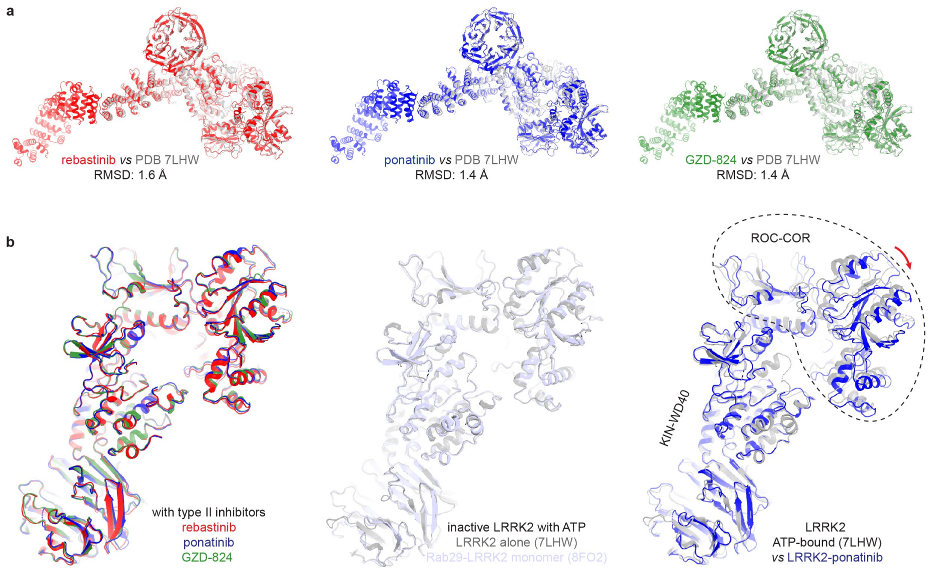

Supplementary Fig. S4 Overall structural comparison between LRRK2 bound to type II inhibitors and the inactive LRRK2. a Structural comparison between LRRK2 in complex with rebastinib (left), ponatinib (middle) or GZD-824 (right) and the inactive LRRK2 (PDB 7LHW). **b** Comparison of LRRK2 structures in complex with different type-II inhibitors (left); comparison between the ATP-bound inactive LRRK2 (PDB 7LHW) and LRRK2 from the inactive Rab29-LRRK2 monomer (middle); and comparison between the ATP-bound inactive LRRK2 (PDB 7LHW) and the ponatinib-bound LRRK2. The movement of ROC-COR domains is indicated by dashed circle with a red arrow.

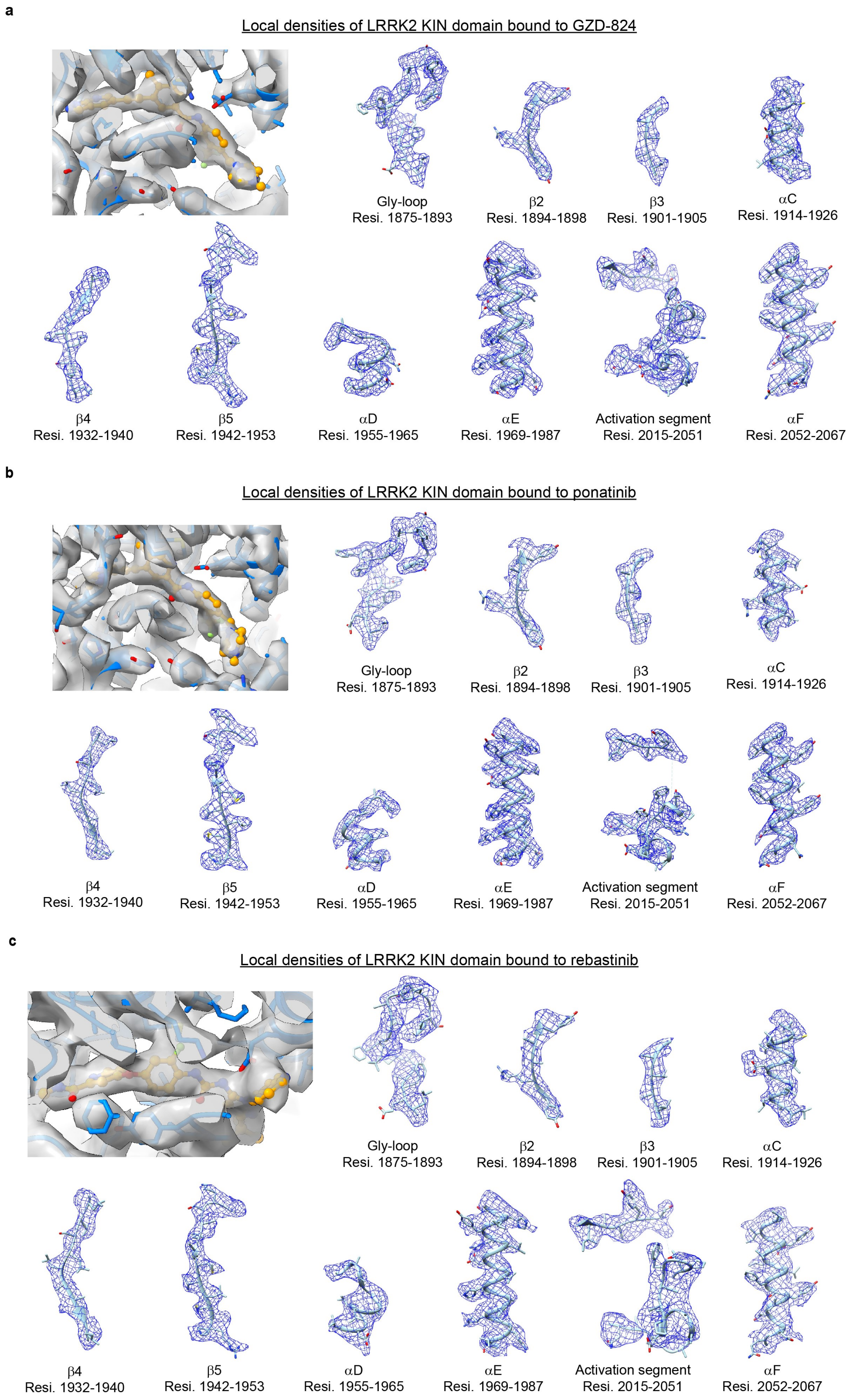

Supplementary Fig. S5 CryoEM density of the inhibitor binding sites and LRRK2 KIN domains from LRRK2-GZD-824 (a), LRRK2-ponatinib (b) and LRRK2-rebastinib (c).

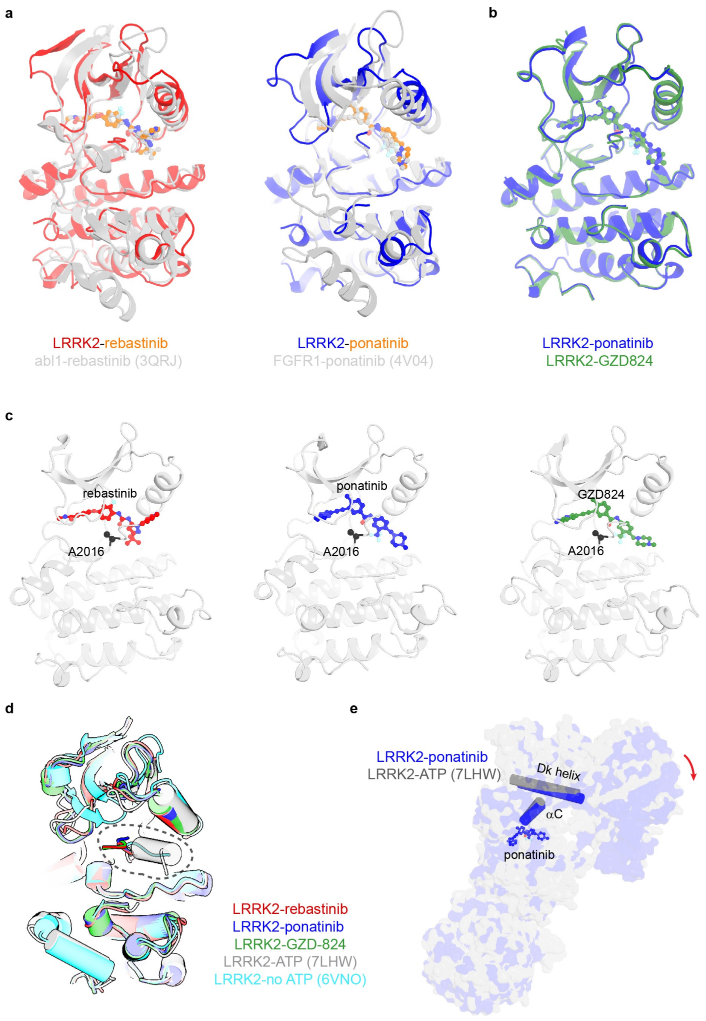

Supplementary Fig. S6 Comparison of the LRRK2 KIN domain with type II inhibitors. a Comparison between LRRK2-rebastinib and Abl1-rebastinib complexes (left), and between LRRK2-ponatinib and FGFR1-ponatinib complexes (right). **b** Overlay of the LRRK2 KIN domain with ponatinib and with GZD-824. **c** Interactions between LRRK2 Ala2016 and rebastinib (left), ponatinib (middle) or GZD-824 (right). **d** Comparison between the LRRK2 KIN domain bound to type-II inhibitors and the inactive LRRK2 KIN domain with or without ATP. A dashed circle indicates the "activation loop" (AL). **e** Comparison between ponatinib-bound LRRK2 and ATP-bound inactive LRRK2 (PDB 7LHW). Movement of the COR-B Dk helix and KIN αC helix upon ponatinib binding is highlighted.

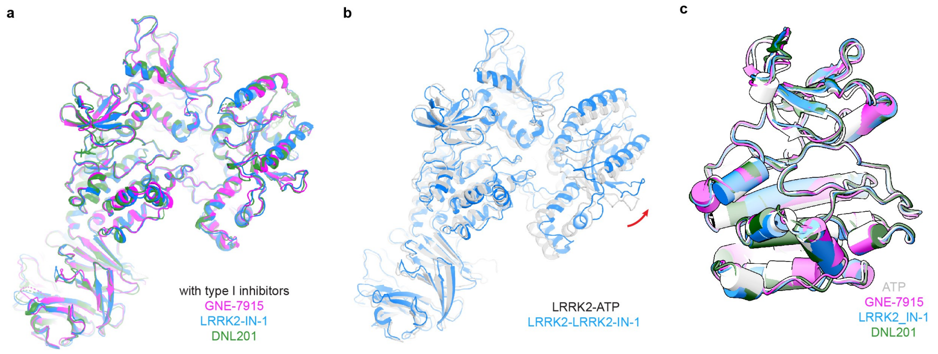

Supplementary Fig. S7 Structural comparison between LRRK2 bound to type I inhibitors with the active LRRK2 from the Rab29-LRRK2 complex. a Overlay of LRRK2 in complex with GNE-7915, LRRK2-IN-1 and DNL201 (PDB 8SMC). **b** Structural comparison of active LRRK2 induced by LRRK2-IN-1 and Rab29 (PDB 8FO9). **c** Overlay of active LRRK2 KIN domains bound to type-I inhibitors and ATP.

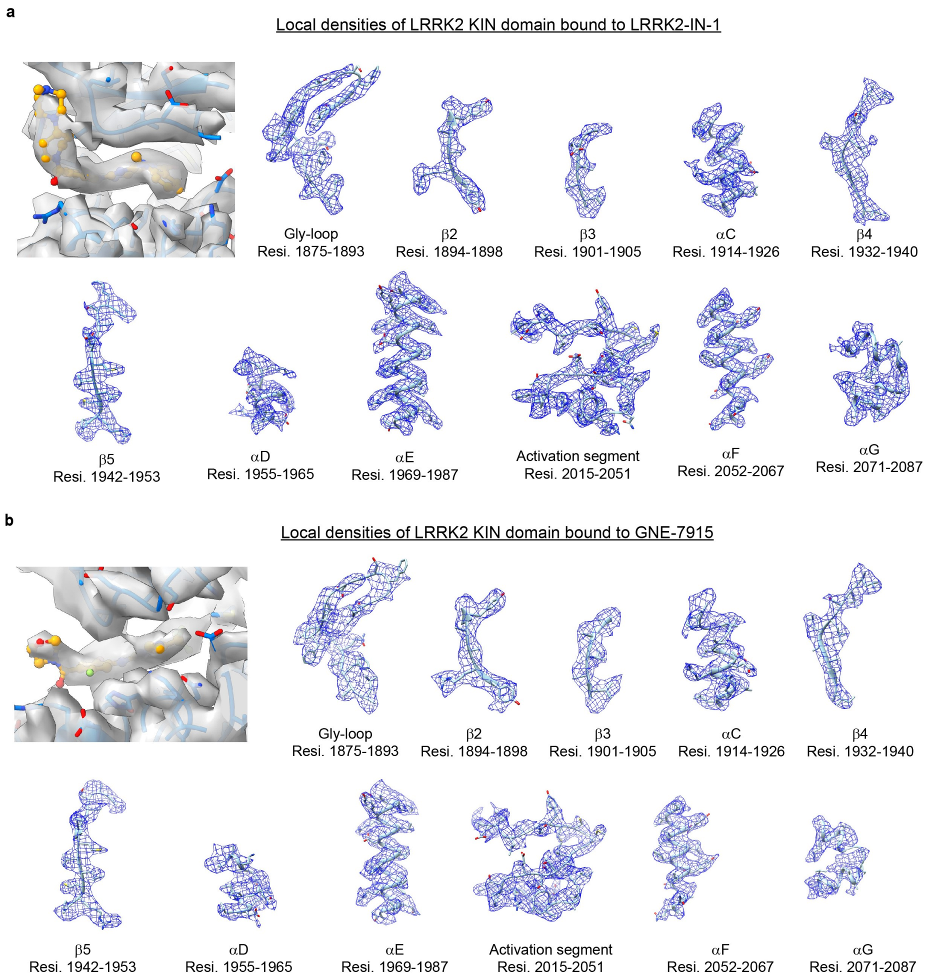

Supplementary Fig. S8 CryoEM density of the inhibitor binding sites and LRRK2 KIN domains from LRRK2-LRRK2-IN-1 (a) and LRRK2-GNE-7915 (b).

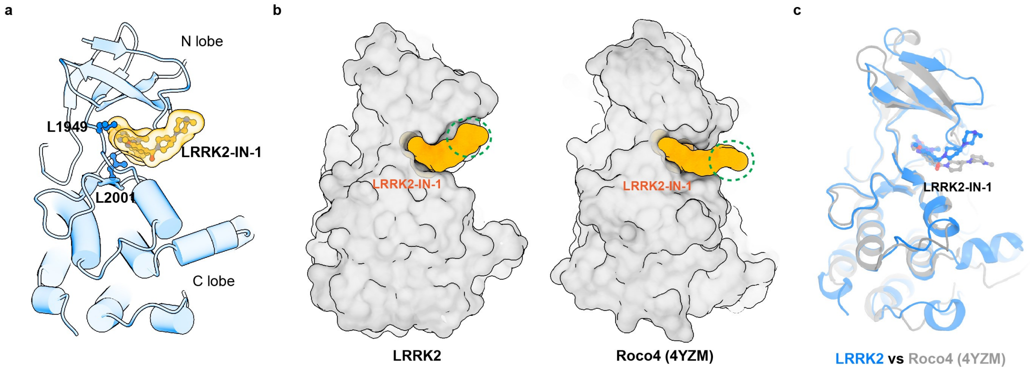

Supplementary Fig. S9 LRRK2-IN-1 binding specificity. a Cartoon model of the LRRK2 KIN domain bound to LRRK2-IN-1. Side chains Leu1949 and Leu2001 are shown, and LRRK2-IN-1 are shown as balls and sticks with transparent surfaces. **b** Structural comparison between LRRK2-LRRK2-IN-1 and and Roco4-LRRK2-IN-1 (PDB 4YZM). The 1-methylpiperazine group of LRRK2-IN-1 is indicated by green dashed circles. **c** Structural comparison between the LRRK2-IN-1-bound KIN domains of LRRK2 and Roco4 (PDB 4YZM).

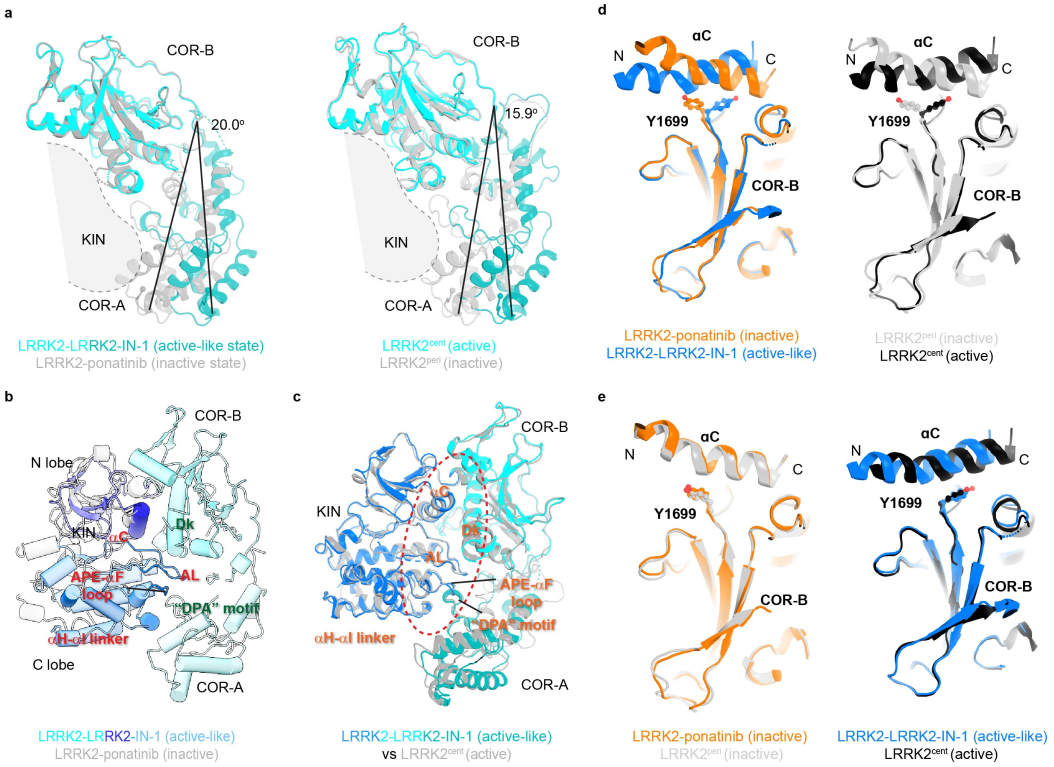

Supplementary Fig. S10 Activation mechanisms of LRRK2 induced by type-I inhibitor and Rab29 binding. a Movement of COR-A subdomain relative to the KIN domain upon the binding of LRRK2-IN-1 (left) and Rab29 (right) (PDB 8FO9). The rotation angles are indicated. **b** Movement of the LRRK2 KIN domain relative to COR domain upon LRRK2-IN-1 binding compared to ponatinib binding. **c** Overlay of KIN-COR domains of LRRK2-IN-1-bound LRRK2 and LRRK2^{cent} in the Rab29-LRRK2 tetramer (PDB 8FO9). The conserved KIN-COR interface is indicated by a brown dashed circle. **d** The seesaw motion of ROC αC helix between LRRK2-ponatinib and LRRK2-LRRK2-IN-1 (left) and between LRRK2^{peri} and LRRK2^{cent} in the Rab29-LRRK2 tetramer (PDB 8FO9). **e** Comparison of the "seesaw" motions in the inactive (LRRK2-ponatinib vs. LRRK2^{peri}) and active (LRRK2-LRRK2-IN-1 vs. LRRK2^{cent}) states.

Supplementary Table S1. Cryo-EM data collection, refinement and validation statistics

	LRRK2–GZD824<br>PDB 8U7L<br>EMD-41985	LRRK2–ponatinib<br>PDB 8U8A<br>EMD-42019	LRRK2–rebastinib<br>PDB 8U8B<br>EMD-42020
**Data collection and processing**			
Microscope/Camera	Titan krios/Gatan K3 Camera		
Magnification	81,000	130,000	130,000
Voltage (kV)	300	300	300
Electron exposure ($e^-/Å^2$)	67.04	68.11	67.53
Defocus range (μm)	0.6-1.8	0.6-1.8	0.6-1.8
Pixel size (Å)	1.06	0.6485	0.6485
Symmetry imposed	C2	C2	C2
Initial particle images (no.)	~2.0 million	~1.9 million	~2.4 million
Final particle images (no.)	78,975	75,849	75,005
Map resolution (Å)	3.6	3.4	3.7
FSC threshold	0.143	0.143	0.143
**Refinement**			
Initial model used (PDB code)	7LI4	7LI4	7LI4
Model resolution (Å)	3.7	3.4	4.0
FSC threshold	0.5	0.5	0.5
Map sharpening *B* factor ($Å^2$)	-122.0	-106.5	-121.6
Model composition			
Non-hydrogen atoms	24,222	24,428	24,694
Protein residue atoms	24,088	24,294	24,556
Ligand atoms	134	134	138
B factors ($Å^2$)			
Protein	99.17	84.19	46.51
Ligand	80.95	49.67	42.90
R.m.s deviations			
Bond lengths (Å)	0.003	0.008	0.003
Bond angles (°)	0.689	0.823	0.770
**Validation**			
MolProbity score	1.80	1.84	1.71
Clashscore	6.03	7.22	4.80
Poor rotamers (%)	0.00	0.17	0.62
Ramachandran plot			
Favored (%)	92.53	93.07	92.66
Allowed (%)	7.47	6.87	7.34
Disallowed (%)	0.00	0.06	0.00

Supplementary Table S2. Cryo-EM data collection, refinement and validation statistics

	LRRK2^{RCKWm}–LRRK2-IN-1<br>PDB 8FO7<br>EMD-29340	LRRK2^{RCKWm}–GNE-7915<br>PDB 8U7H<br>EMD-41982
**Data collection and processing**		
Microscope/Camera	Titan krios/Gatan K3 Camera	
Magnification	130,000	130,000
Voltage (kV)	300	300
Electron exposure (e^-/Å^2)	62.54	62.08
Defocus range (μm)	0.6-2.0	0.8-2.4
Pixel size (Å)	0.6485	0.6485
Symmetry imposed	C1	C1
Initial particle images (no.)	~1.2 million	~4.2 million
Final particle images (no.)	187,407	95,644
Map resolution (Å)	3.5	3.8
FSC threshold	0.143	0.143
**Refinement**		
Initial model used (PDB code)	8FO9	8FO7
Model resolution (Å)	3.7	3.8
FSC threshold	0.5	0.5
Map sharpening *B* factor (Å^2)	-130.0	-119.1
Model composition		
Non-hydrogen atoms	8,452	7,867
Protein residue atoms	8,382	7,808
Ligand atoms	70	59
B factors (Å^2)		
Protein	27.43	68.49
Ligand	43.52	31.16
R.m.s deviations		
Bond lengths (Å)	0.003	0.003
Bond angles (°)	0.684	0.835
**Validation**		
MolProbity score	1.62	1.82
Clashscore	5.20	6.30
Poor rotamers (%)	0.12	0.14
Ramachandran plot		
Favored (%)	95.10	92.09
Allowed (%)	4.90	7.91
Disallowed (%)	0.00	0.00

Supplementary Video S1: Conformation transition revealed by MD simulations.
